# (*S*)-3-Bromo-4-diallyl­amino-5-[(1*R*,2*S*,5*R*)-2-isopropyl-5-methyl­cyclo­hex­yloxy]furan-2(5*H*)-one

**DOI:** 10.1107/S1600536810047173

**Published:** 2010-11-24

**Authors:** Jian-Hua Fu, Zhao-Yang Wang, Jian-Xiao Li, Yue-He Tan

**Affiliations:** a School of Chemistry and Environment, South China Normal University, Guangzhou 510006, People’s Republic of China

## Abstract

The title compound, C_20_H_30_BrNO_3_, was obtained *via* a tandem asymmetric Michael addition–elimination reaction of 3,4-dibromo-5-(*S*)-(l-menth­yloxy)-2(5*H*)-furan­one and diallyl­amine in the presence of potassium fluoride. In the mol­ecule, the five-membered furan­one ring is approximately planar [maximum atomic deviation = 0.030 (3) Å], and the six-membered cyclo­hexane ring adopts a chair conformation.

## Related literature

The title compound is a derivative of 4-amino-2(5*H*)-furan­one. For the biological activity of 4-amino-2(5*H*)-furan­ones, see: Gondela & Walczak (2010 ▶); Tanoury *et al.* (2008 ▶); Kimura *et al.* (2000 ▶). For asymmetric Michael addition reactions of 2(5*H*)-furan­one, see: Hoffmann *et al.* (2006 ▶); He *et al.* (2006) ▶. For the synthesis of the title compound, see: Song *et al.* (2009 ▶).
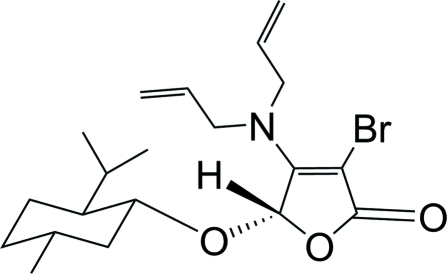


## Experimental

### 

#### Crystal data

C_20_H_30_BrNO_3_
*M* *_r_* = 412.35
Orthorhombic, 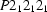

*a* = 8.5215 (16) Å
*b* = 11.934 (2) Å
*c* = 20.603 (4) Å
*V* = 2095.2 (7) Å^3^
*Z* = 4
Mo *K*α radiation
μ = 1.98 mm^−1^
*T* = 298 K
0.23 × 0.20 × 0.16 mm

#### Data collection

Bruker APEXII area-detector diffractometer
Absorption correction: multi-scan (*SADABS*; Sheldrick, 1996 ▶) *T*_min_ = 0.641, *T*_max_ = 0.729
19608 measured reflections
3640 independent reflections
2660 reflections with *I* > 2σ(*I*)
*R*_int_ = 0.078

#### Refinement

*R*[*F* ^2^ > 2σ(*F* ^2^)] = 0.037
*wR*(*F* ^2^) = 0.075
*S* = 1.04
3640 reflections
230 parameters
H-atom parameters constrained
Δρ_max_ = 0.28 e Å^−3^
Δρ_min_ = −0.21 e Å^−3^
Absolute structure: Flack (1983 ▶), 1543 Friedel pairs
Flack parameter: 0.001 (9)

### 

Data collection: *APEX2* (Bruker, 2008 ▶); cell refinement: *SAINT* (Bruker, 2008 ▶); data reduction: *SAINT*; program(s) used to solve structure: *SHELXS97* (Sheldrick, 2008 ▶); program(s) used to refine structure: *SHELXL97* (Sheldrick, 2008 ▶); molecular graphics: *ORTEP-3 for Windows* (Farrugia, 1997 ▶); software used to prepare material for publication: *SHELXL97*.

## Supplementary Material

Crystal structure: contains datablocks global, I. DOI: 10.1107/S1600536810047173/xu5089sup1.cif

Structure factors: contains datablocks I. DOI: 10.1107/S1600536810047173/xu5089Isup2.hkl

Additional supplementary materials:  crystallographic information; 3D view; checkCIF report

## Figures and Tables

**Fig. 1.**
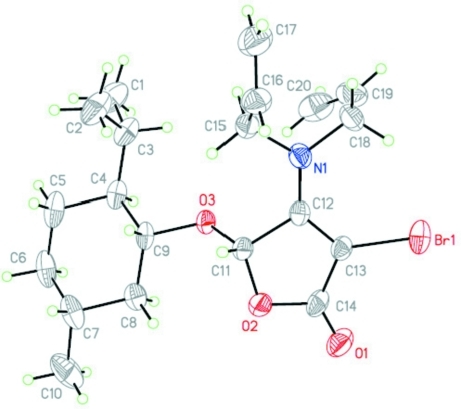
The molecular structure of the title compound showing the atom-labelling scheme. Ellipsoids are drawn at the 50% probability level.

**Fig. 2.**
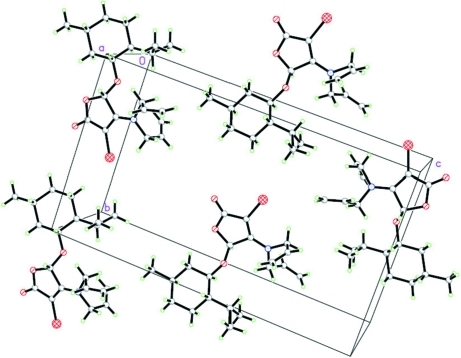
Perspective view of the crystal packing.

## supplementary crystallographic information

### Comment

The 2(5H)-furanone moiety occurs in many natural products exhibiting various biological activities, namely antibiotic cytotoxic and antitumor (Gondela et al., 2010). Recently, owing to their specific activity and high stereoselectivity, chiral 5-S-(l-menthyloxy)-2(5H)-furanones have emerged as significant synthetic intermediates (Hoffmann et al., 2006; Song et al., 2009). At the same time, 4-amino-2(5H)-furanone (or 3-amino-2(5H)-furanone) is an attractive moiety in chemical, pharmaceutical and agrochemical research (Tanoury et al., 2008; Kimura et al., 2000).

Therefore we are interested in the tandem Michael addition-elimination reaction of the chiral synthon 3,4-dibromo-5-(S)-(l-menthyloxy)-2(5H)-furanone and diallylamine in the present of potassium fluoride. The structure of the title compound (I) is illustrated in Fig. 1. The crystal structure of the title compound which has four chiral centers (C11(S), C9(R), C4(S), C7(R)) contains a five-membered furanone ring and a six-membered rings connected each other via C11—O3—C9 ether bond. The furanone ring of C11—O2—C14—C13—C12 is approximately planar, whereas the six-membered ring displays a chair conformation.

### Experimental

The precursor 3,4-dibromo-5-(S)-(l-menthyloxy)-2(5H)-furanone was prepared according to the literature procedure (Song et al., 2009). After the mixture of 3,4-dibromo-5-(S)-(l-menthyloxy)-2(5H)-furanone (2.0 mmol) and potassium fluoride (6.0 mmol) was dissolved in absolute tetrahydrofuran (2.0 mL) under nitrogen atmosphere, tetrahydrofuran solution of diallylamine (3.0 mmol) was added. The reaction was carried out under the stirring at room temperature for 24 h. Once the reaction was complete, the solvents were removed under reduced pressure. The residual solid was dissolved in dichloromethane. Then the combined organic layers from extraction were concentrated under reduced pressure, and the crude product was purified by silica gel column chromatography with the gradient mixture of petroleum ether and ethyl acetate to give the product yielding (I) 0.645 g (78.3%).

### Refinement

H atoms were positioned in calculated positions with C—H = 0.93-0.98 Å and were refined using a riding model, with U_iso_(H) = 1.5U_eq_(C) for methyl and 1.2U_eq_(C) for the others.

### Crystal data

**Table:**

C_20_H_30_BrNO_3_	*F*(000) = 864
*M**_r_* = 412.35	*D*_x_ = 1.307 Mg m^−^^3^
Orthorhombic, *P*2_1_2_1_2_1_	Mo *K*α radiation, λ = 0.71073 Å
Hall symbol: P 2ac 2ab	Cell parameters from 3500 reflections
*a* = 8.5215 (16) Å	θ = 2.6–20.9°
*b* = 11.934 (2) Å	µ = 1.98 mm^−^^1^
*c* = 20.603 (4) Å	*T* = 298 K
*V* = 2095.2 (7) Å^3^	Block, colourless
*Z* = 4	0.23 × 0.20 × 0.16 mm

### Data collection

**Table:**

Bruker APEXII area-detector diffractometer	3640 independent reflections
Radiation source: fine-focus sealed tube	2660 reflections with *I* > 2σ(*I*)
graphite	*R*_int_ = 0.078
φ and ω scan	θ_max_ = 24.9°, θ_min_ = 2.0°
Absorption correction: multi-scan (*SADABS*; Sheldrick, 1996)	*h* = −10→10
*T*_min_ = 0.641, *T*_max_ = 0.729	*k* = −14→14
19608 measured reflections	*l* = −24→24

### Refinement

**Table:**

Refinement on *F*^2^	Secondary atom site location: difference Fourier map
Least-squares matrix: full	Hydrogen site location: inferred from neighbouring sites
*R*[*F*^2^ > 2σ(*F*^2^)] = 0.037	H-atom parameters constrained
*wR*(*F*^2^) = 0.075	*w* = 1/[σ^2^(*F*_o_^2^) + (0.*P*)^2^ + 0.1072*P*] where *P* = (*F*_o_^2^ + 2*F*_c_^2^)/3
*S* = 1.04	(Δ/σ)_max_ < 0.001
3640 reflections	Δρ_max_ = 0.28 e Å^−^^3^
230 parameters	Δρ_min_ = −0.21 e Å^−^^3^
0 restraints	Absolute structure: Flack (1983), 1543 Friedel pairs
Primary atom site location: structure-invariant direct methods	Flack parameter: 0.001 (9)

### Special details

**Table:**

Geometry. All esds (except the esd in the dihedral angle between two l.s. planes) are estimated using the full covariance matrix. The cell esds are taken into account individually in the estimation of esds in distances, angles and torsion angles; correlations between esds in cell parameters are only used when they are defined by crystal symmetry. An approximate (isotropic) treatment of cell esds is used for estimating esds involving l.s. planes.
Refinement. Refinement of F^2^ against ALL reflections. The weighted R-factor wR and goodness of fit S are based on F^2^, conventional R-factors R are based on F, with F set to zero for negative F^2^. The threshold expression of F^2^ > 2sigma(F^2^) is used only for calculating R-factors(gt) etc. and is not relevant to the choice of reflections for refinement. R-factors based on F^2^ are statistically about twice as large as those based on F, and R- factors based on ALL data will be even larger.

### Fractional atomic coordinates and isotropic or equivalent isotropic displacement parameters (Å^2^)

**Table:**

	*x*	*y*	*z*	*U*_iso_*/*U*_eq_	
Br1	0.27706 (5)	−0.04364 (3)	0.92085 (2)	0.07258 (18)	
O3	0.2972 (3)	0.36270 (16)	0.96994 (9)	0.0435 (5)	
O2	0.3680 (3)	0.21670 (19)	1.03849 (10)	0.0574 (7)	
O1	0.2726 (3)	0.0470 (2)	1.06339 (12)	0.0797 (8)	
C4	0.2198 (4)	0.5548 (3)	0.97362 (17)	0.0567 (9)	
H4	0.1129	0.5295	0.9834	0.068*	
C9	0.3298 (4)	0.4689 (3)	1.00221 (16)	0.0479 (9)	
H9	0.4381	0.4911	0.9927	0.057*	
C8	0.3107 (4)	0.4579 (3)	1.07491 (17)	0.0579 (8)	
H8A	0.3878	0.4051	1.0911	0.070*	
H8B	0.2074	0.4277	1.0842	0.070*	
C6	0.2203 (5)	0.6563 (3)	1.0818 (2)	0.0822 (12)	
H6A	0.1126	0.6350	1.0907	0.099*	
H6B	0.2394	0.7284	1.1021	0.099*	
C3	0.2302 (4)	0.5667 (3)	0.89958 (18)	0.0670 (11)	
H3	0.2262	0.4906	0.8819	0.080*	
C5	0.2427 (5)	0.6670 (3)	1.0096 (2)	0.0798 (13)	
H5A	0.3476	0.6949	1.0010	0.096*	
H5B	0.1685	0.7214	0.9928	0.096*	
C2	0.3854 (5)	0.6182 (4)	0.8759 (2)	0.0923 (14)	
H2A	0.3909	0.6952	0.8892	0.138*	
H2B	0.3903	0.6140	0.8294	0.138*	
H2C	0.4719	0.5776	0.8943	0.138*	
C10	0.3028 (6)	0.5538 (4)	1.1835 (2)	0.1184 (18)	
H10A	0.1984	0.5261	1.1906	0.178*	
H10B	0.3154	0.6243	1.2054	0.178*	
H10C	0.3774	0.5009	1.2003	0.178*	
C7	0.3301 (4)	0.5697 (3)	1.1109 (2)	0.0727 (12)	
H7	0.4382	0.5956	1.1046	0.087*	
C12	0.3881 (3)	0.1916 (2)	0.92585 (17)	0.0411 (7)	
C11	0.4052 (4)	0.2769 (3)	0.98021 (15)	0.0441 (8)	
H11	0.5124	0.3065	0.9819	0.053*	
C13	0.3321 (4)	0.0962 (3)	0.95381 (17)	0.0468 (9)	
C14	0.3192 (4)	0.1112 (3)	1.02266 (19)	0.0557 (10)	
N1	0.4230 (3)	0.2185 (2)	0.86442 (14)	0.0512 (8)	
C16	0.6441 (5)	0.3143 (3)	0.81261 (19)	0.0658 (11)	
H16	0.7254	0.2830	0.8366	0.079*	
C17	0.6743 (6)	0.3435 (3)	0.7539 (2)	0.0898 (14)	
H17A	0.5960	0.3751	0.7283	0.108*	
H17B	0.7744	0.3329	0.7369	0.108*	
C15	0.4897 (4)	0.3267 (3)	0.84513 (18)	0.0547 (10)	
H15A	0.5020	0.3734	0.8833	0.066*	
H15B	0.4175	0.3642	0.8159	0.066*	
C18	0.3789 (5)	0.1460 (3)	0.81058 (17)	0.0659 (11)	
H18A	0.3965	0.0687	0.8231	0.079*	
H18B	0.4467	0.1620	0.7739	0.079*	
C19	0.2127 (6)	0.1588 (4)	0.7897 (2)	0.0828 (13)	
H19	0.1814	0.1184	0.7534	0.099*	
C20	0.1081 (6)	0.2206 (4)	0.8173 (2)	0.0991 (16)	
H20A	0.1340	0.2626	0.8538	0.119*	
H20B	0.0067	0.2232	0.8006	0.119*	
C1	0.0902 (5)	0.6287 (4)	0.8715 (2)	0.0987 (16)	
H1A	0.0917	0.7051	0.8861	0.148*	
H1B	−0.0049	0.5934	0.8857	0.148*	
H1C	0.0954	0.6269	0.8250	0.148*	

### Atomic displacement parameters (Å^2^)

**Table:**

	*U*^11^	*U*^22^	*U*^33^	*U*^12^	*U*^13^	*U*^23^
Br1	0.0696 (3)	0.0426 (2)	0.1055 (3)	−0.0069 (2)	0.0069 (2)	−0.0098 (2)
O3	0.0464 (13)	0.0334 (11)	0.0508 (13)	0.0051 (11)	0.0006 (11)	−0.0065 (10)
O2	0.0797 (18)	0.0465 (14)	0.0460 (15)	0.0092 (13)	0.0040 (13)	0.0029 (12)
O1	0.1046 (19)	0.0580 (15)	0.0766 (18)	0.0109 (18)	0.0256 (16)	0.0173 (14)
C4	0.050 (2)	0.0383 (18)	0.082 (3)	−0.001 (2)	0.008 (2)	−0.0055 (17)
C9	0.0412 (19)	0.0343 (18)	0.068 (3)	−0.0073 (16)	0.0054 (16)	−0.0098 (17)
C8	0.055 (2)	0.054 (2)	0.065 (2)	0.0018 (19)	0.002 (2)	−0.011 (2)
C6	0.071 (3)	0.054 (2)	0.122 (4)	−0.005 (2)	0.011 (3)	−0.039 (2)
C3	0.071 (3)	0.046 (2)	0.084 (3)	0.004 (2)	0.012 (2)	0.0154 (17)
C5	0.073 (3)	0.040 (2)	0.126 (4)	0.000 (2)	0.007 (3)	−0.012 (2)
C2	0.094 (4)	0.068 (3)	0.115 (4)	0.001 (3)	0.031 (3)	0.028 (3)
C10	0.141 (4)	0.129 (4)	0.085 (4)	0.017 (4)	0.002 (3)	−0.060 (3)
C7	0.057 (2)	0.071 (3)	0.090 (3)	−0.001 (2)	0.007 (2)	−0.039 (2)
C12	0.0338 (17)	0.0386 (18)	0.051 (2)	0.0041 (13)	0.0021 (18)	−0.0023 (18)
C11	0.046 (2)	0.0382 (18)	0.048 (2)	0.0053 (16)	0.0020 (17)	−0.0017 (17)
C13	0.048 (2)	0.0388 (19)	0.053 (2)	0.0103 (16)	0.0032 (17)	−0.0003 (16)
C14	0.055 (3)	0.039 (2)	0.073 (3)	0.0175 (18)	0.015 (2)	0.0080 (19)
N1	0.0573 (19)	0.0478 (17)	0.048 (2)	−0.0038 (15)	0.0100 (15)	−0.0076 (15)
C16	0.070 (3)	0.067 (3)	0.061 (3)	−0.006 (2)	0.006 (2)	0.009 (2)
C17	0.107 (4)	0.082 (3)	0.081 (3)	0.005 (3)	0.018 (3)	0.021 (2)
C15	0.065 (3)	0.046 (2)	0.054 (2)	−0.0049 (18)	0.007 (2)	−0.0010 (17)
C18	0.084 (3)	0.064 (3)	0.050 (2)	−0.014 (2)	0.010 (2)	−0.015 (2)
C19	0.086 (3)	0.097 (3)	0.065 (3)	−0.024 (3)	−0.017 (3)	0.003 (2)
C20	0.070 (3)	0.125 (4)	0.103 (4)	−0.011 (3)	−0.018 (3)	0.037 (4)
C1	0.099 (4)	0.086 (3)	0.111 (4)	0.017 (3)	0.003 (3)	0.030 (3)

### Geometric parameters (Å, °)

**Table:**

Br1—C13	1.862 (3)	C10—H10A	0.9600
O3—C11	1.393 (4)	C10—H10B	0.9600
O3—C9	1.458 (3)	C10—H10C	0.9600
O2—C14	1.365 (4)	C7—H7	0.9800
O2—C11	1.434 (4)	C12—N1	1.339 (4)
O1—C14	1.204 (4)	C12—C13	1.362 (4)
C4—C9	1.509 (4)	C12—C11	1.521 (4)
C4—C3	1.535 (5)	C11—H11	0.9800
C4—C5	1.542 (4)	C13—C14	1.434 (5)
C4—H4	0.9800	N1—C18	1.456 (4)
C9—C8	1.512 (4)	N1—C15	1.466 (4)
C9—H9	0.9800	C16—C17	1.285 (5)
C8—C7	1.535 (5)	C16—C15	1.484 (5)
C8—H8A	0.9700	C16—H16	0.9300
C8—H8B	0.9700	C17—H17A	0.9300
C6—C5	1.505 (5)	C17—H17B	0.9300
C6—C7	1.518 (5)	C15—H15A	0.9700
C6—H6A	0.9700	C15—H15B	0.9700
C6—H6B	0.9700	C18—C19	1.488 (6)
C3—C1	1.518 (5)	C18—H18A	0.9700
C3—C2	1.538 (5)	C18—H18B	0.9700
C3—H3	0.9800	C19—C20	1.288 (6)
C5—H5A	0.9700	C19—H19	0.9300
C5—H5B	0.9700	C20—H20A	0.9300
C2—H2A	0.9600	C20—H20B	0.9300
C2—H2B	0.9600	C1—H1A	0.9600
C2—H2C	0.9600	C1—H1B	0.9600
C10—C7	1.526 (6)	C1—H1C	0.9600
			
C11—O3—C9	116.3 (2)	C10—C7—C8	110.4 (3)
C14—O2—C11	109.2 (3)	C6—C7—H7	108.2
C9—C4—C3	114.5 (3)	C10—C7—H7	108.2
C9—C4—C5	108.9 (3)	C8—C7—H7	108.2
C3—C4—C5	112.9 (3)	N1—C12—C13	132.7 (3)
C9—C4—H4	106.7	N1—C12—C11	120.9 (3)
C3—C4—H4	106.7	C13—C12—C11	106.3 (3)
C5—C4—H4	106.7	O3—C11—O2	110.5 (3)
O3—C9—C4	107.1 (2)	O3—C11—C12	108.5 (2)
O3—C9—C8	110.9 (3)	O2—C11—C12	105.1 (2)
C4—C9—C8	112.3 (3)	O3—C11—H11	110.9
O3—C9—H9	108.8	O2—C11—H11	110.9
C4—C9—H9	108.8	C12—C11—H11	110.9
C8—C9—H9	108.8	C12—C13—C14	109.9 (3)
C9—C8—C7	113.1 (3)	C12—C13—Br1	133.1 (3)
C9—C8—H8A	109.0	C14—C13—Br1	117.0 (2)
C7—C8—H8A	109.0	O1—C14—O2	121.4 (3)
C9—C8—H8B	109.0	O1—C14—C13	129.4 (3)
C7—C8—H8B	109.0	O2—C14—C13	109.2 (3)
H8A—C8—H8B	107.8	C12—N1—C18	121.3 (3)
C5—C6—C7	111.8 (3)	C12—N1—C15	123.6 (3)
C5—C6—H6A	109.3	C18—N1—C15	114.7 (3)
C7—C6—H6A	109.3	C17—C16—C15	125.1 (4)
C5—C6—H6B	109.3	C17—C16—H16	117.4
C7—C6—H6B	109.3	C15—C16—H16	117.4
H6A—C6—H6B	107.9	C16—C17—H17A	120.0
C1—C3—C4	112.2 (3)	C16—C17—H17B	120.0
C1—C3—C2	111.1 (3)	H17A—C17—H17B	120.0
C4—C3—C2	113.7 (3)	N1—C15—C16	112.2 (3)
C1—C3—H3	106.4	N1—C15—H15A	109.2
C4—C3—H3	106.4	C16—C15—H15A	109.2
C2—C3—H3	106.4	N1—C15—H15B	109.2
C6—C5—C4	112.7 (3)	C16—C15—H15B	109.2
C6—C5—H5A	109.1	H15A—C15—H15B	107.9
C4—C5—H5A	109.1	N1—C18—C19	113.9 (4)
C6—C5—H5B	109.1	N1—C18—H18A	108.8
C4—C5—H5B	109.1	C19—C18—H18A	108.8
H5A—C5—H5B	107.8	N1—C18—H18B	108.8
C3—C2—H2A	109.5	C19—C18—H18B	108.8
C3—C2—H2B	109.5	H18A—C18—H18B	107.7
H2A—C2—H2B	109.5	C20—C19—C18	126.2 (5)
C3—C2—H2C	109.5	C20—C19—H19	116.9
H2A—C2—H2C	109.5	C18—C19—H19	116.9
H2B—C2—H2C	109.5	C19—C20—H20A	120.0
C7—C10—H10A	109.5	C19—C20—H20B	120.0
C7—C10—H10B	109.5	H20A—C20—H20B	120.0
H10A—C10—H10B	109.5	C3—C1—H1A	109.5
C7—C10—H10C	109.5	C3—C1—H1B	109.5
H10A—C10—H10C	109.5	H1A—C1—H1B	109.5
H10B—C10—H10C	109.5	C3—C1—H1C	109.5
C6—C7—C10	112.2 (3)	H1A—C1—H1C	109.5
C6—C7—C8	109.5 (3)	H1B—C1—H1C	109.5
			
C11—O3—C9—C4	168.3 (2)	C13—C12—C11—O3	−113.1 (3)
C11—O3—C9—C8	−68.9 (3)	N1—C12—C11—O2	−176.3 (3)
C3—C4—C9—O3	−56.2 (3)	C13—C12—C11—O2	5.0 (3)
C5—C4—C9—O3	176.3 (3)	N1—C12—C13—C14	178.5 (3)
C3—C4—C9—C8	−178.1 (3)	C11—C12—C13—C14	−3.0 (4)
C5—C4—C9—C8	54.4 (4)	N1—C12—C13—Br1	0.3 (6)
O3—C9—C8—C7	−175.2 (3)	C11—C12—C13—Br1	178.8 (3)
C4—C9—C8—C7	−55.4 (4)	C11—O2—C14—O1	−175.5 (3)
C9—C4—C3—C1	164.3 (3)	C11—O2—C14—C13	3.6 (4)
C5—C4—C3—C1	−70.3 (4)	C12—C13—C14—O1	178.8 (3)
C9—C4—C3—C2	−68.5 (4)	Br1—C13—C14—O1	−2.6 (5)
C5—C4—C3—C2	56.9 (4)	C12—C13—C14—O2	−0.2 (4)
C7—C6—C5—C4	57.1 (4)	Br1—C13—C14—O2	178.3 (2)
C9—C4—C5—C6	−55.9 (4)	C13—C12—N1—C18	10.4 (5)
C3—C4—C5—C6	175.7 (3)	C11—C12—N1—C18	−167.9 (3)
C5—C6—C7—C10	−177.1 (3)	C13—C12—N1—C15	−177.2 (3)
C5—C6—C7—C8	−54.0 (4)	C11—C12—N1—C15	4.5 (5)
C9—C8—C7—C6	53.5 (4)	C12—N1—C15—C16	120.4 (3)
C9—C8—C7—C10	177.6 (3)	C18—N1—C15—C16	−66.7 (4)
C9—O3—C11—O2	85.9 (3)	C17—C16—C15—N1	117.8 (4)
C9—O3—C11—C12	−159.4 (2)	C12—N1—C18—C19	80.9 (4)
C14—O2—C11—O3	111.6 (3)	C15—N1—C18—C19	−92.2 (4)
C14—O2—C11—C12	−5.2 (3)	N1—C18—C19—C20	−4.7 (7)
N1—C12—C11—O3	65.6 (3)		

### Hydrogen-bond geometry (Å, °)

**Table:**

*D*—H···*A*	*D*—H	H···*A*	*D*···*A*	*D*—H···*A*
C20—H20A···N1	0.93	2.53	2.854 (6)	101
C18—H18A···Br1	0.97	2.62	3.322 (4)	129
C15—H15A···O3	0.97	2.50	3.081 (4)	118
C8—H8A···O2	0.97	2.50	3.015 (4)	113
C3—H3···O3	0.98	2.45	2.891 (4)	107
